# The mechanism of the premetastatic niche facilitating colorectal cancer liver metastasis generated from myeloid-derived suppressor cells induced by the S1PR1–STAT3 signaling pathway

**DOI:** 10.1038/s41419-019-1922-5

**Published:** 2019-09-18

**Authors:** Qi Lin, Li Ren, Mi Jian, Pingping Xu, Jun Li, Peng Zheng, Qingyang Feng, Liangliang Yang, Meilin Ji, Ye Wei, Jianmin Xu

**Affiliations:** Department of General Surgery, Zhongshan Hospital, Fudan University, 180 Fenglin Rd, Shanghai, 200032 China

**Keywords:** Cancer microenvironment, Colon cancer

## Abstract

The tumor-derived factors involved in the expansion and accumulation of myeloid-derived suppressor cells (MDSCs) in metastatic dissemination of colorectal cancer (CRC) to the liver has not been studied. Immunohistochemistry was used to detect sphingosine-1-phosphate receptor 1 (S1PR1) and signal transducer and activator of transcription-3 (STAT3) in human colorectal tumors. IL-6 and interferon-γ were detected by enzyme-linked immunosorbent assay (ELISA). Tumor growth, invasion, and migration were evaluated by MTT, transwell, and wound healing assays, respectively. Subcutaneous tumor-bearing and CRC liver metastasis (CRLM) nude mouse models were constructed. The percentage of MDSCs was measured using multicolor flow cytometry. Western blot assay was used to evaluate S1PR1 and p-STAT3 expression in MDSCs after separation from the liver and tumor by magnetic antibody. T-cell suppression assay was detected by carboxyfluorescein succinimidyl ester (CFSE). Aberrant co-expressed S1PR1 and p-STAT3 was correlated with metachronous liver metastasis and poor prognosis in CRC. A mutual activation loop between S1PR1 and STAT3 can enhance CRC cell proliferation, migration, and invasion in vitro and in vivo. The expression of p-STAT3 and its downstream proteins can be regulated by S1PR1. p-STAT3 was the dependent signaling pathway of S1PR1 in the promotion of cell growth and liver metastasis in CRC. The level of IL-6 and the associated MDSCs stimulated by the S1PR1–STAT3 correlated with the number of liver metastatic nodes in the CRLM mouse models and patients. Increased CD14^+^HLA-DR^−/low^ MDSCs from CRLM patients inhibited autologous T-cell proliferation and predict poor prognosis. The S1PR1–STAT3–IL-6–MDSCs axis operates in both tumor cells and MDSCs involved in the promotion of growth and liver metastasis in CRC. MDSCs induced by S1PR1–STAT3 in CRC cells formed the premetastatic niche in the liver can promote organ-specific metastasis.

## Introduction

Approximately, 25% of patients with colorectal cancer (CRC) present with liver metastases at initial diagnosis and almost 50% will develop liver metastases. Even after radical resection, relapse can occur in 75% of patients, and 50% of relapses are in the liver^1^, contributing to the high mortality rates reported for CRC^1^. Therefore, development of new treatment modalities for colorectal liver metastasis (CRLM) is urgently required and a greater understanding of the biology of the liver metastatic process will help establish new therapeutics aimed at prevention and treatment of CRLM.

Assemblages of ostensibly normal tissue and bone marrow-derived (stromal) cells are recruited to constitute tumorigenic microenvironments, which are the accessories to tumor metastasis. This enhanced understanding may present interesting new targets for anticancer therapy^2^.

An intriguing study recently identified sphingosine-1-phosphate receptor 1 (S1PR1) as a key element involved in the persistent activation of signal transducer and activator of transcription-3 (STAT3) both in tumor cells and the tumor microenvironment in animal models of melanoma (B16) and bladder cancer (MB49). Enhanced S1PR1 expression activates STAT3 and upregulates IL-6 expression, a proinflammatory cytokine crucial for STAT3 activation and inflammatory cell-mediated transformation and tumor progression^3^. IL-6 is a multifunctional cytokine important for immune responses, cell survival, apoptosis, and proliferation^4^. IL-6 is also known to be an important mediator of the expansion and recruitment of myeloid-derived suppressor cells (MDSCs)^5,6^. MDSCs is a heterogeneous population of immature hematopoietic cells comprised of the monocyte or granulocyte lineage that expand dramatically under conditions such as trauma, tumor growth, and various chronic inflammatory disorders, including infection, sepsis, and immunization. These cells have a role in immune tolerance, tumor progression, and metastasis^5,6^. Tumor progression is associated with increased numbers of MDSCs in the primary tumor microenvironment and circulation, which in turn is associated with a poor prognosis, supporting a role in metastasis^7^. Recently, cancer (such as breast, melanoma, and bladder) derived remote signals were shown to induce the accumulation of myeloid cells including MDSCs populations in putative metastatic sites before migrating cancer cells arrived, forming a “premetastatic niche” which aided extravasation of migrating cancer cells and facilitated new blood vessel formation^8,9^. However, the tumor-derived factors involved in the expansion and accumulation of MDSCs in the metastatic dissemination of CRC to the liver has not been studied.

Previously, we reported that the expression of S1PR1 was significantly upregulated in 70.6% (108/153) of the CRC primary lesions and was correlated with metachronous liver metastasis^10^. Based on the above background, we speculated that a S1PR1–STAT3–IL-6–MDSCs signaling pathway may be involved in the promotion of growth and liver metastasis in CRC and that MDSCs could form a “premetastatic niche” for the CRC liver-specific metastasis.

## Results

### Aberrant co-expressed S1PR1 and p-STAT3 was correlated with metachronous liver metastasis and poor prognosis in CRC

**Fig. 1:**
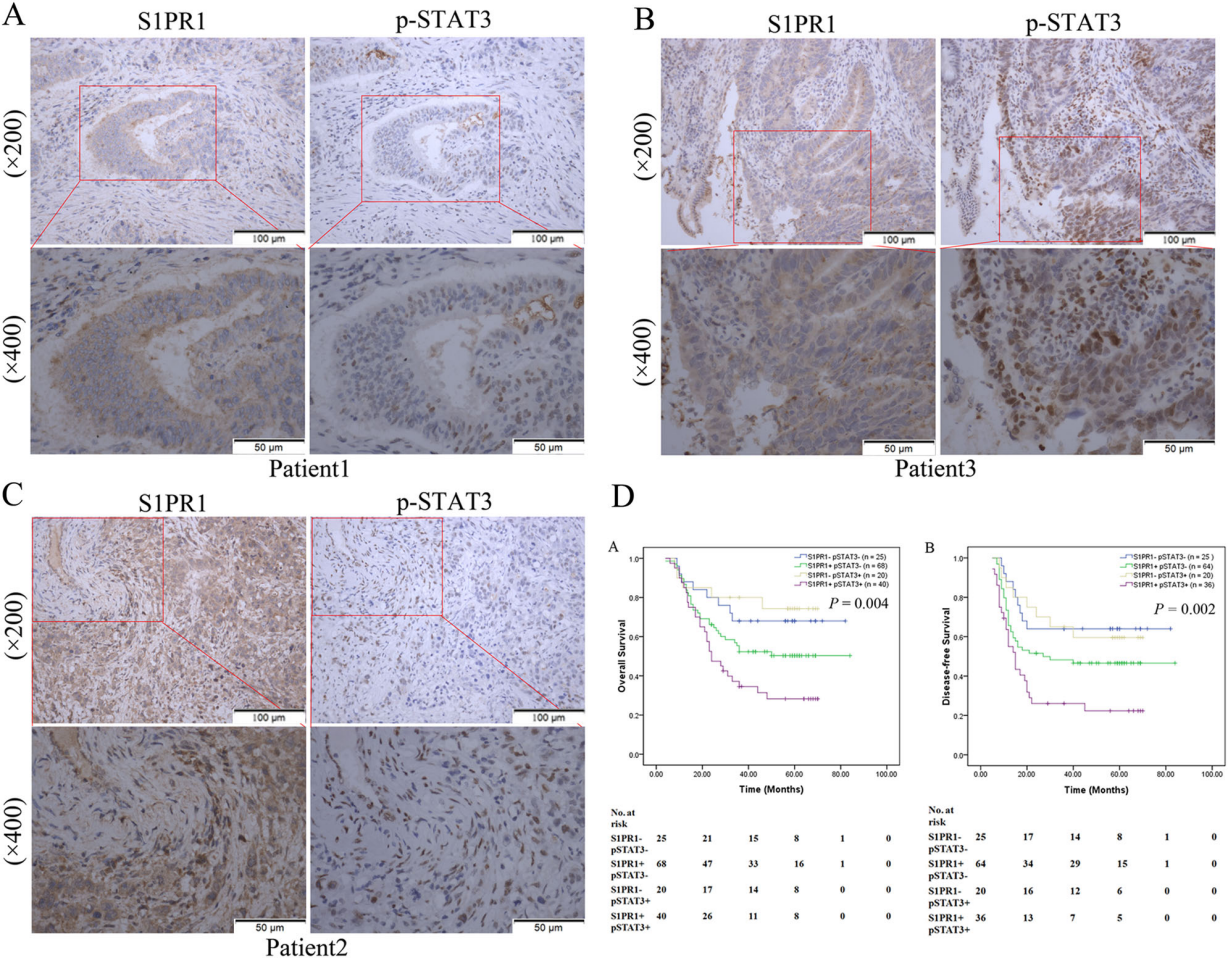
Immunohistochemical staining of S1PR1 and p-STAT3 and analyses of survival. **a–c** Representative photomicrographs of S1PR1 and p-STAT3 of the same region of three patients, using consecutive patient tissue sections. Scale bar: 50.0 μm, 100 μm. **d** Kaplan–Meier analyses of overall survival and disease-free survival according to the expression of S1PR1 and p-STAT3 (aberrant overlapping expression or low expression)

As a transcriptional factor, STAT3 plays a crucial role in promoting the progression of human cancers, including CRC, and is associated with adverse clinical outcome^11,12^. Presently, we reported that the expression of S1PR1 was significantly upregulated in 70.6% (108/153) of the CRC lesions compared to high expression only in 5.9% (9/153) of the adjacent noncancerous tissues (*P* < 0.001)^10^. In this study, we evaluated S1PR1 and p-STAT3 expression simultaneously by immunohistochemical analyses with consecutive sections. We found that the expression of p-STAT3 was significantly upregulated in 39.2% (60/153) of the CRC lesions while the adjacent noncancerous tissues had almost no expression. S1PR1 and p-STAT3 were highly co-expressed in 26.1% (40/153) of the CRC lesions (Fig. 1a–c), and 37.5% (15/40) of these patients developed metachronous liver metastasis. As shown in Table 1, aberrant co-expressed of S1PR1 and p-STAT3 was not correlated with any clinicopathological factors, except for a positive correlation with metachronous liver metastasis (*P* = 0.022). As shown in Table 2, univariate analyses determined that vascular invasion (*P* = 0.035), T classification (*P* *=* 0.035), N classification (*P* < 0.001), M classification (*P* < 0.01), CEA level (*P* = 0.024), and aberrant co-expressed S1PR1 and p-STAT3 (*P* = 0.002) were statistically significant risk factors affecting the overall survival (OS) of these patients. To evaluate the robustness of the prognostic value, Cox multivariate regression analysis was performed to derive independent risk estimates related to OS with the covariates showing significance in univariate analyses. As shown in Table 3, N classification (*P* = 0.001), M classification (*P* = 0.005), and aberrant co-expressed S1PR1 and p-STAT3 (*P* = 0.007) were recognized as independent prognostic factors for poor OS. As shown in Fig. 1d, the Kaplan–Meier survival analysis revealed that the OS and disease-free survival (DFS) of CRC patients with aberrant co-expressed S1PR1 and p-STAT3 were significantly poorer than those patients with low expression (*P* = 0.004, *P* *=* 0.002, respectively).

**Table 1:** Relation between intratumoral S1PR1 and p-STAT3 co-expression and clinical characteristics in patients with CRC

Variables	Patients		S1PR1 and p-STAT3		*P* value
	No.	%	Co-expression	Not co-expression	
	153	100	40	113	
*Age (years)*					0.807
≤ 60	97	63.4	26	71	
> 60	56	36.6	14	42	
*Gender*					0.212
Male	78	51.0	17	61	
Female	75	49.0	23	52	
*Tumor location*					0.164
Colon	87	56.9	19	68	
Rectum	66	43.1	21	45	
*Tumor size*					0.659
≤ 5 cm	58	37.9	26	69	
> 5 cm	95	62.1	14	44	
*Gross appearance*					0.820
Ulcerative	113	73.9	29	84	
Exophytic	40	26.1	11	29	
*Histological type*					0.795
Adenocarcinoma	136	88.9	36	100	
Mucinous adenocarcinoma	17	11.1	4	13	
*Tumor differentiation*					0.113
Well, moderate	54	35.3	10	44	
Poor and others	99	64.7	30	69	
*Depth of invasion*					0.283
T1	11	7.2	5	6	
T2	29	18.9	7	22	
T3	39	25.5	7	32	
T4	74	48.4	21	53	
*Vascular invasion*					0.405
Absent	135	88.2	37	98	
Present	18	11.8	3	15	
*Lymph node metastasis*					0.781
N0	60	39.2	14	46	
N1	54	35.3	16	38	
N2	39	25.5	10	29	
M					0.207
M0	145	94.8	36	109	
M1	8	5.2	4	4	
*Metachronous liver metstassis*					0.022
Absent	116	75.8	25	91	
Present	37	24.2	15	22	
*TNM stage*					0.439
I	20	13.1	4	16	
II	39	25.5	9	30	
III	86	56.2	23	63	
IV	8	5.2	4	4	
*CEA*					0.502
≤ 5 ng/ml	91	59.5	22	69	
>5 ng/ml	62	40.5	18	44

**Table 2:** Univariate analysis of clinicopathological factors for OS in 153 CRC patients

Characteristics	OS	
	Exp(B) (95% CI)	*P*
Age: >60 vs. ≤60	0.876 (0.543–1.411)	0.585
Gender: female vs. male	0.931 (0.590–1.469)	0.758
Tumor location: rectum vs. colon	1.110 (0.702–1.756)	0.656
Tumor size > 5 cm vs. ≤5 cm	1.024 (0.642–1.633)	0.920
Gross appearance: exophytic vs. ulcerative	0.689 (0.396–1.199)	0.188
Histological type: mucinous adenocarcinoma vs. adenocarcinoma	1.291 (0.662–2.517)	0.453
Tumor differentiation: poor and others vs. well, moderate	1.556 (0.944–2.563)	0.083
Vascular invasion: positive vs. negative	1.908 (1.046–3.478)	0.035
T: T3,4 vs. T1,2	1.873 (1.046–3.354)	0.035
Lymph node metastasis: positive vs. negative	2.953 (1.715–5.083)	<0.001
M: M1 vs. M0	4.450 (2.100–9.431)	<0.001
CEA: >5 ng/ml vs. ≤5 ng/ml	1.694 (1.074–2.672)	0.024
p-STAT3: positive vs. negative	1.324 (0.837–2.095)	0.230
S1PR1 and p-STAT3: co-expression vs. not co-expression	2.085 (1.300–3.343)	0.002

**Table 3:** Multivariate Cox regression model for OS in 153 CRC patients

Characteristics	OS	
	Exp(B) (95% CI)	*P*
Vascular invasion: positive vs. negative	1.604 (0.852–3.021)	0.143
T: T3,4 vs. T1,2	1.629 (0.898–2.955)	0.108
Lymph node metastasis: positive vs. negative	2.567 (1.471–4.478)	0.001
M: M1 vs. M0	3.044 (1.409–6.577)	0.005
CEA: >5 ng/ml vs. ≤5 ng/ml	1.352 (0.841–2.174)	0.214
S1PR1 and p-STAT3: co-expression vs. not co-expression	1.944 (1.197–3.155)	0.007

### S1PR1 can promote CRC cell proliferation, invasion, migration, and liver metastasis in vitro and in vivo

**Fig. 2:**
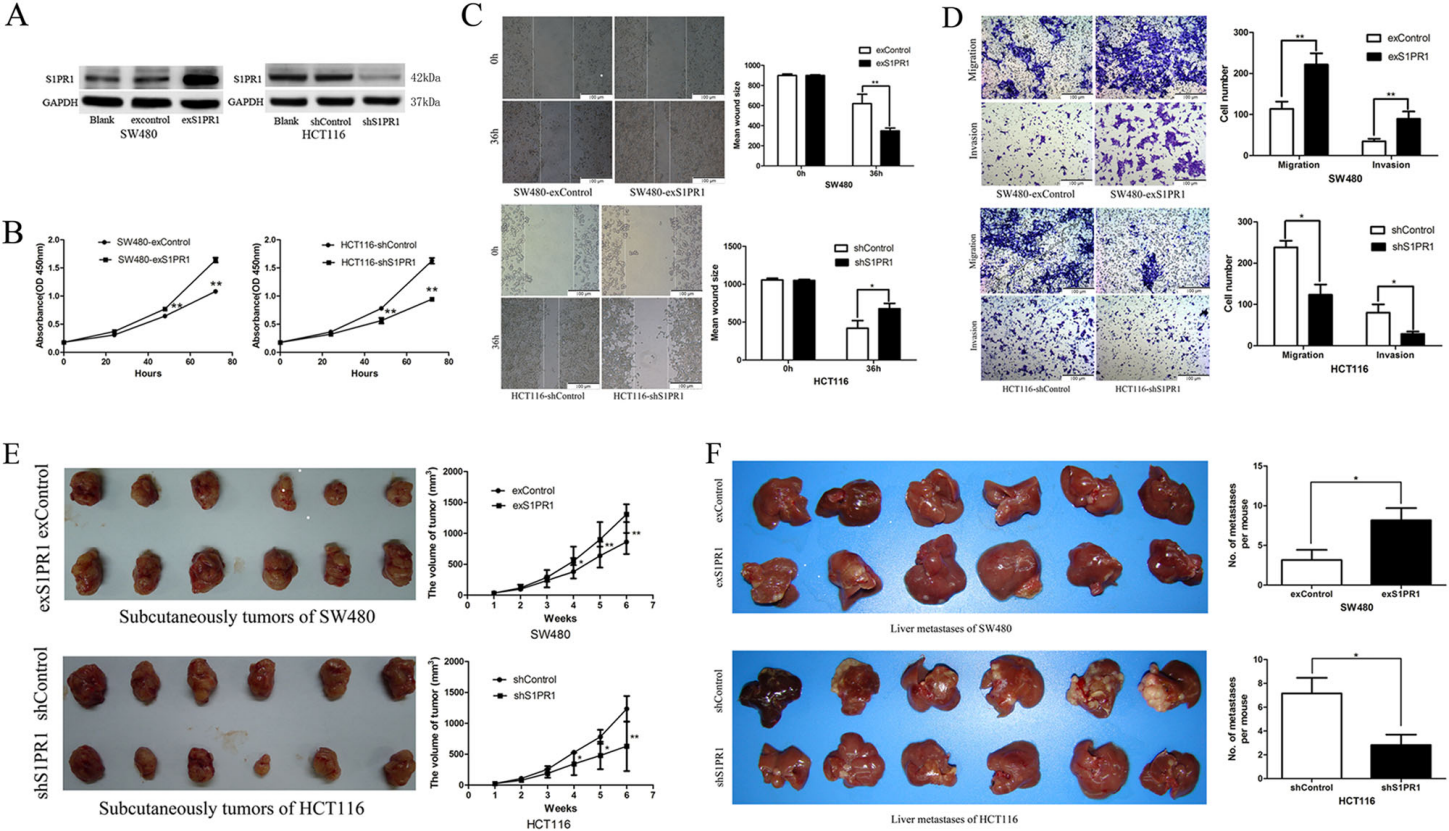
S1PR1 promote CRC cell proliferation, invasion, migration and liver metastasis in vitro and in vivo. **a** S1PR1 protein expression levels in SW480 cells transduced with lenti-exS1PR1 and in HCT116 cells transduced with lenti-shS1PR1, as revealed using Western blotting. **b** S1PR1 overexpression significantly promoted the growth of SW480 and S1PR1 knockdown significantly inhibited the growth of HCT116, as revealed using an MTT assay. **c** A wound healing assay showed S1PR1 overexpression significantly promoted cell migration of SW480 and S1PR1 knockdown significantly inhibited the migration of HCT116, 36 h after wounding. **d** A transwell assay showed S1PR1 overexpression significantly promoted cell migration and invasion of SW480 and S1PR1 knockdown significantly inhibited the migration and invasion of HCT116. **e** The morphological characteristics of subcutaneous tumors with S1PR1 overexpression in SW480 and S1PR1 knockdown in HCT116. **f** Characteristic images of liver metastases after S1PR1 overexpression in SW480 and S1PR1 knockdown in HCT116. The data shown represent the mean ± s.e.m of a representative experiment performed in triplicate (^*^P < 0.05, ^**^P < 0.01)

Because the clinical data showed a positive correlation between S1PR1 expression and metastatic behavior in CRC^10^, the roles of *S1PR1* in proliferation, invasion, and migration were investigated in the SW480 and HCT116 human CRC cell lines. S1PR1 protein was lowly expressed in SW480 and highly expressed in HCT116 (Supplementary Fig. 1). We then introduced the pCDH-EF1-MCS-IRES-GFP-S1PR1 plasmid and lenti-shRNA targeting S1PR1 into SW480 and HCT116, respectively. Both the S1PR1 overexpression and shRNA lentivirus vectors were successfully transfected (Fig. 2a). We found that S1PR1 overexpression significantly promoted cell proliferation, as determined using an MTT assay (Fig. 2b), cell migration, as determined using a wound healing assay (Fig. 2c), and cell invasion, as determined using a transwell assay (Fig. 2d). Then, the impact of S1PR1 on CRC cell proliferation in vivo was analyzed by subcutaneously inoculating tumors into nude mice. S1PR1 overexpression resulted in a significant increase in tumor size compared with control tumors (Fig. 2e). The impact of S1PR1 on CRC cell metastasis was then investigated following xenotransplantation into nude mice through intrasplenic injection, and we found that the number of distant masses was significantly increased in S1PR1 overexpression tumor cells at 6 weeks post injection (Fig. 2f). After S1PR1 expression was significantly downregulated in HCT116, the proliferation (Fig. 2b), migration (Fig. 2c), and invasion (Fig. 2d) capabilities were significantly decreased in vitro when compared with the control group and resulted in a significant decrease in subcutaneous tumor size (Fig. 2e) and fewer liver metastases in vivo (Fig. 2f).

### Targeting S1PR1 can regulate STAT3 and the STAT3-mediated signaling pathway

**Fig. 3:**
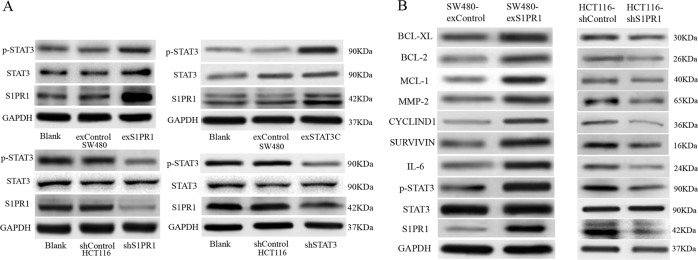
Targeting S1PR1 can regulate the STAT3-mediated signaling pathway. **a** Western blot analysis of protein expression levels of S1PR1 and p-STAT3 in SW480 and HCT116 after overexpression or shRNA with S1PR1 and STAT3, respectively. **b** Western blot analysis of protein expression levels of STAT3 downstream genes in SW480 and HCT116 after overexpression or shRNA with S1PR1 and STAT3, respectively

In the tumor cell lines of the activated B cell-like subtype of diffuse large B-cell lymphoma, inhibition of S1PR1 expression by shRNA in the lymphoma cells validated that blocking S1PR1 affects the expression of STAT3 and STAT3 downstream genes critically involved in tumor cell survival, proliferation, tumor invasion, and/or immunosuppression^13^. We, therefore, investigated whether targeting S1PR1 can regulate the STAT3-mediated signaling pathway in CRC. First, using Western blot analysis, we found that the expression of p-STAT3 was concordantly expressed with S1PR1 and that S1PR1 and p-STAT3 were co-expressed in SW480 and HCT116 after overexpression or shRNA with S1PR1 and STAT3, respectively (Fig. 3a). Furthermore, we found that the level of p-STAT3 and its downstream proteins, including IL-6, CYCLIND1, MCL-1, BCL-2, BCL-XL, SURVIVIN, and MMP-2, were increased after S1PR1 overexpression and decreased after S1PR1 shRNA (Fig. 3b).

### P-STAT3 was required for S1PR1-promoted tumor growth and liver metastasis in CRC

**Fig. 4:**
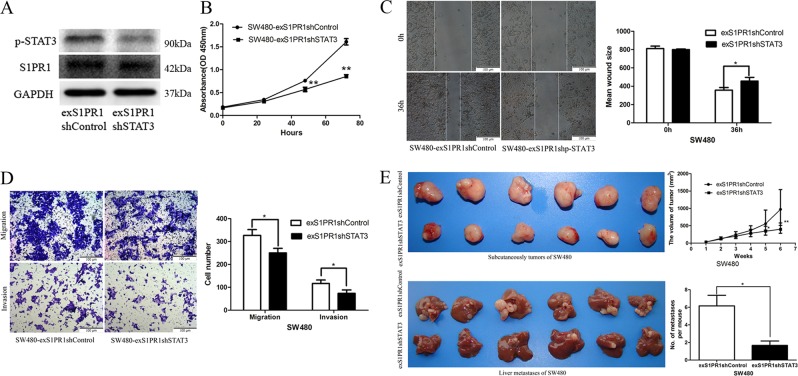
P-STAT3 is required for S1PR1-promoted tumor growth and liver metastasis in CRC. **a** S1PR1 and p-STAT3 protein expression levels in SW480-exS1PR1 cells transduced with lenti-shSTAT3, as revealed using Western blotting. **b** STAT3 knockdown significantly inhibited the growth of SW480-exS1PR1 cells, as revealed using an MTT assay. **c** A wound healing assay showed STAT3 knockdown significantly inhibited the migration of SW480-exS1PR1 cells, 36 h after wounding. **d** A transwell assay showed STAT3 knockdown significantly inhibited cell migration and invasion of SW480-exS1PR1. **e** The morphologic characteristics of subcutaneous tumors in STAT3 knockdown SW480-exS1PR1 cells; characteristic images of liver metastases in STAT3 knockdown SW480-exS1PR1 cells. The data shown represent the mean ± s.e.m of a representative experiment performed in triplicate (^*^P < 0.05, ^**^P < 0.01)

Given that targeting S1PR1 can regulate the STAT3-mediated signaling pathway in our study and others^13^, we examined whether inhibiting STAT3 could constrain S1PR1-facilitated cell growth and liver metastasis. We used lentiviral-based delivery of shSTAT3 to deplete p-STAT3 expression in the SW480-exS1PR1 cells (Fig. 4a). By cell proliferation assay, wound healing and transwell assay in vitro and subcutaneous tumor-bearing and CRLM nude mouse models in vivo, we found that downregulated p-STAT3 in SW480-exS1PR1 cells resulted in significantly decreased proliferation (Fig. 4b), migration (Fig. 4c), and invasion (Fig. 4d) in vitro, when compared with the control group, and a significant decrease in subcutaneous tumor size and liver metastases in vivo (Fig. 4e).

### Level of IL-6 and the associated MDSCs stimulated by the S1PR1–STAT3 signaling pathway correlate with the number of liver metastatic nodes in the CRLM mouse model

Aberrant IL-6-STAT3 signaling in cancer cells has emerged as an important mechanism for cancer initiation, progression and metastasis^14,15^. Enhanced S1PR1 expression activates STAT3 and upregulates *Il6* gene expression in MB49 tumor cells^3^. IL-6 is a downstream mediator of the cytokine-induced expansion of MDSCs^5,6^. Furthermore, in Fig. 3b, we found that IL-6 changed most significantly among p-STAT3 downstream proteins. Therefore, we investigated whether more IL-6 is generated in the CRLM mouse model after activating the S1PR1–STAT3 signaling pathway, whether IL-6 can recruit more MDSCs, and whether IL-6-associated MDSCs correlate with the number of liver metastatic nodes.

**Fig. 5:**
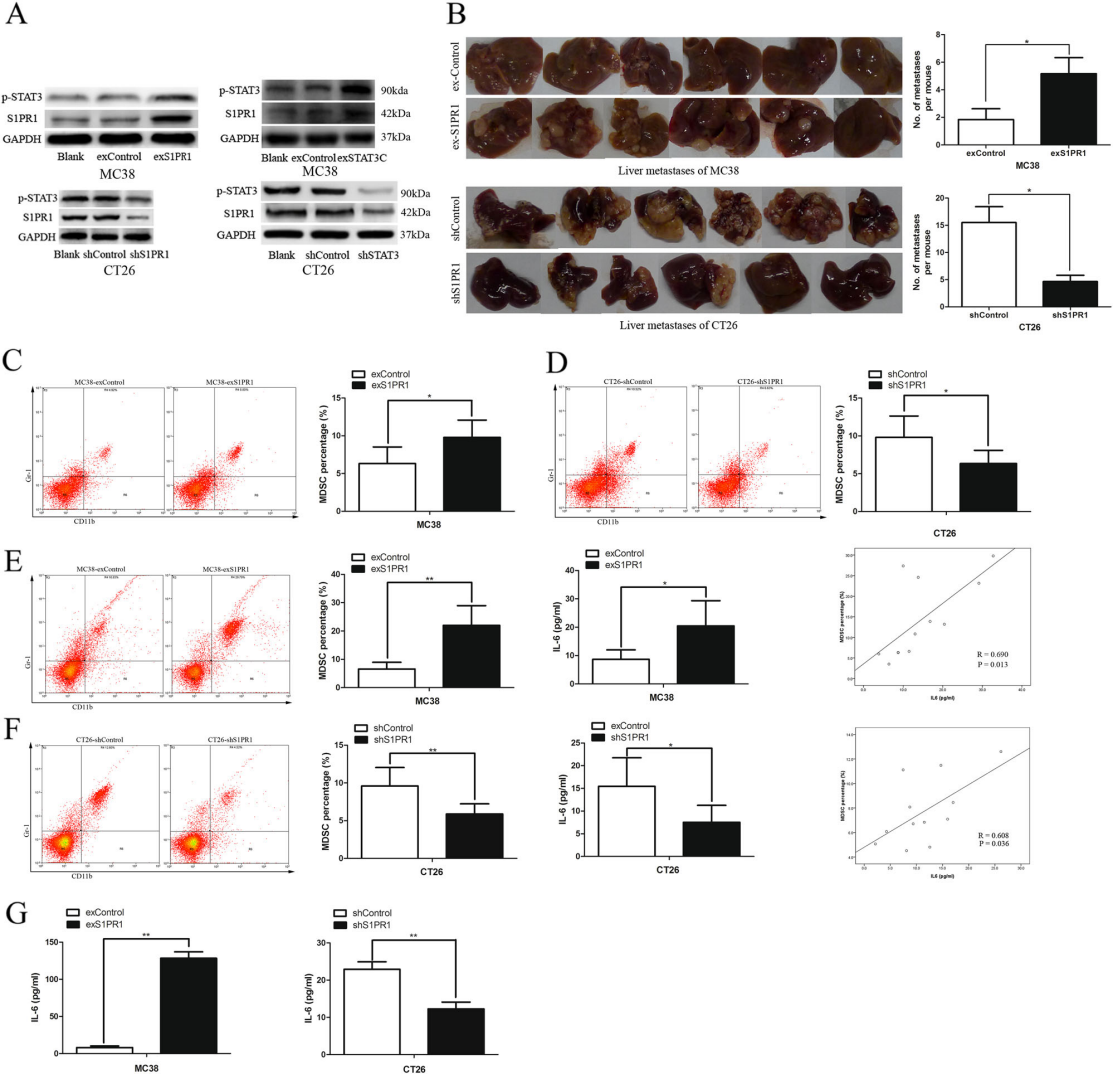
IL-6 and associated MDSCs stimulated by S1PR1–STAT3 correlated with liver metastatic nodes numbers in vivo. **a** S1PR1 and p-STAT3 were co-expressed in MC38 and CT26 after overexpression or shRNA with S1PR1 and STAT3, respectively. **b** Characteristic images of liver metastases from S1PR1 overexpression in MC38 and S1PR1 knockdown in CT26. **c**, **d** The levels of the MDSCs of the paracancer liver tissue of the MC38 and CT26 CRLM mouse models. **e**, **f** The levels of the MDSCs and IL-6 in the blood of the MC38 and CT26 CRLM mouse models, respectively, and the correlation between them analyzed by correlation analysis. **g** The levels of IL-6 in the cell culture supernatant of the MC38 cell line with S1PR1 overexpression and CT26 cell line with S1PR1 knockdown. The data shown represent the mean ± s.e.m of a representative experiment performed in triplicate (^*^P < 0.05, ^**^P < 0.01)

To investigate the tumor microenvironment, the murine derived CRC cell lines MC38 and CT26 were used. S1PR1 protein was lowly expressed in MC38 and highly expressed in CT26 cells (Supplementary Fig. 2). Both overexpression and shRNA lentivirus vectors were successfully transfected into MC38 and CT26 cells, respectively. Similar to the human cell lines SW480 and HCT116, using a Western blot assay, we found that S1PR1 and p-STAT3 were co-expressed in MC38 and CT26 after overexpression or shRNA with S1PR1 and STAT3, respectively (Fig. 5a).

The metastatic liver nodes (Fig. 5b), the percentage of MDSCs in the paracancer liver (Fig. 5c), the level of circulating IL-6 and MDSCs in the blood (Fig. 5e) and IL-6 in the cell culture supernatant (Fig. 5g) in the MC38-exS1PR1 group were higher than the MC38-exControl group. The metastatic liver nodes (Fig. 5b), the percentage of MDSCs in the paracancer liver (Fig. 5d), the level of circulating IL-6 and MDSCs in the blood (Fig. 5f) and cell culture supernatant (Fig. 5g) in the CT26-shS1PR1 group were lower than the CT26-shControl group. Furthermore, we found that the level of circulating IL-6 and MDSCs in the blood were positively correlated in the MC38 and CT26 liver metastatic models, respectively (Fig. 5e, f). These results demonstrated that in the CRLM model, the level of IL-6 and the associated MDSCs stimulated by the S1PR1–STAT3 signaling pathway was positively correlated with the number of liver metastatic nodes. We also found the MDSCs from paracancer liver tissue of the CRLM mice models (both MC38 and CT26) significantly inhibited the T-cell proliferation compared to that from the control liver tissue (Supplementary Fig. 3A, B). The gating strategy for mice MDSCs was showed in the Supplementary Fig. 4C.

### S1PR1–STAT3-induced tumor factors activate S1PR1–STAT3 in MDSCs, which form the premetastatic niche in the liver to promote CRLM

**Fig. 6:**
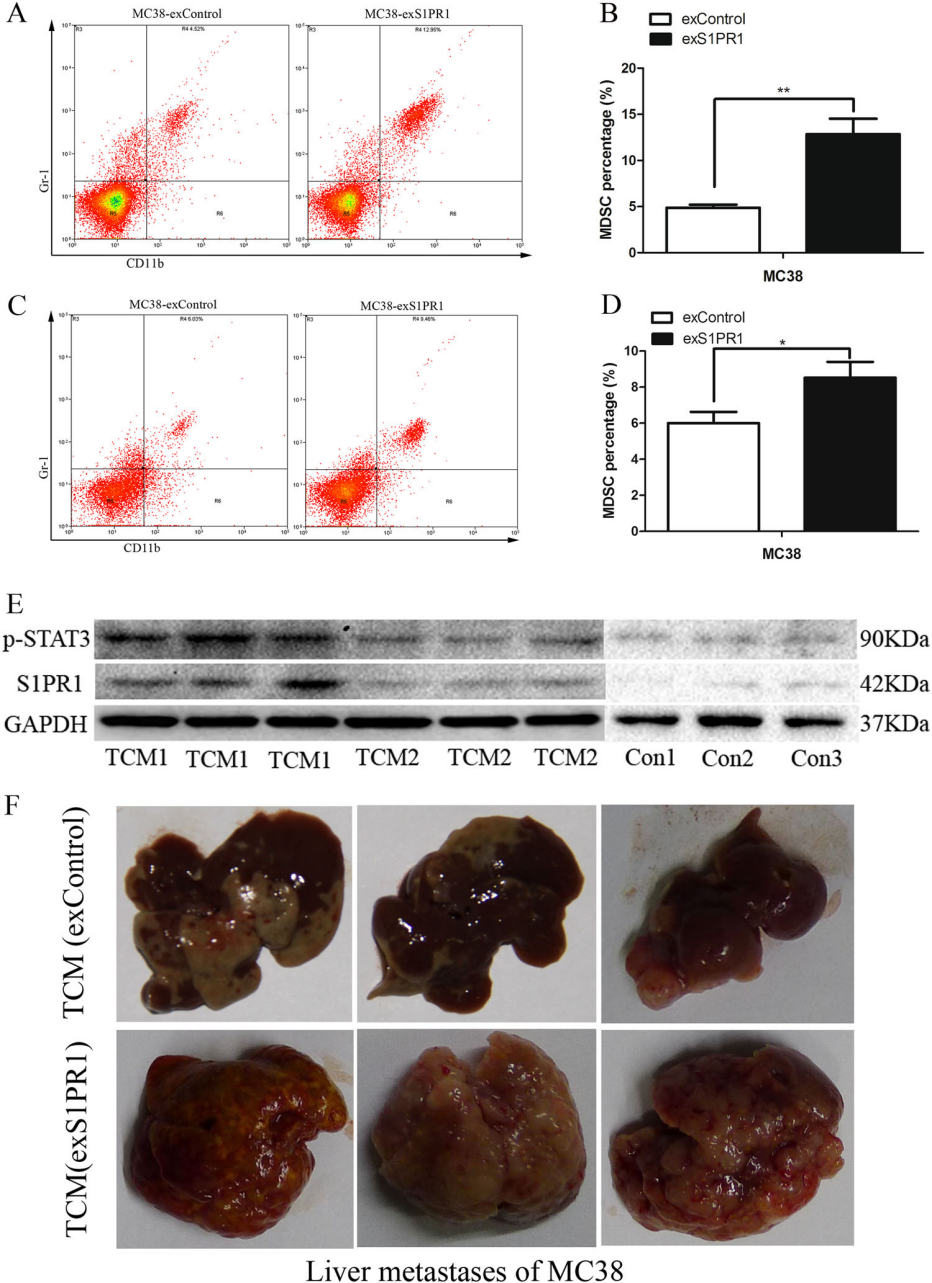
S1PR1–STAT3-induced tumor factors activate S1PR1–STAT3 in MDSCs, forming premetastatic niche in the liver promoting CRLM. **a**, **b** Treatment with TCM derived from the MC38-exS1PR1 group induced more MDSC infiltration in the blood and compared to the control group. **c**, **d** Treatment with TCM derived from the MC38-exS1PR1 group induced more MDSC infiltration in the paracancer liver tissue compared to the control group. **e** Treatment with TCM derived from the MC38-exS1PR1 group induced strong S1PR1 and p-STAT3 activation in MDSCs without tumor cell challenge compared to the control group and untreated group. **f** Treatment with TCM derived from MC38-exS1PR1 could induce extensive metastasis at 21 days post tumor cell challenge. The data shown represent the mean ± s.e.m of a representative experiment performed in triplicate (^*^P < 0.05, ^**^P < 0.01)

To investigate whether increased S1PR1–STAT3 signaling in CRC cells would induce a production of factors that could prime distant premetastatic sites in the liver, we generated tumor conditioned media (TCM) from the MC38-exS1PR1 group and MC38-exControl group. We found that IL-6 in the cell culture supernatant of the MC38-exS1PR1 group was higher than the MC38-exControl group (Fig. 5g). In the absence of tumor cell challenge, treatment with TCM derived from the MC38-exS1PR1 group and control for 7 days, when there were no detectable metastases, led to more MDSCs infiltration in the blood (Fig. 6a, b) and paracancer liver (Fig. 6c, d) of the MC38-exS1PR1 group compared to the control group. We also found treatment with TCM derived from the MC38-exS1PR1 group induced strong S1PR1 and p-STAT3 activation in MDSCs without tumor cell challenge compared to the MC38-exControl group and untreated group (Fig. 5e). Furthermore, treating mice with TCM generated from MC38-exS1PR1, but not TCM derived from control groups, could induce extensive metastasis 21 days post tumor cell challenge (Fig. 6f).

### High level of circulating IL-6 associated with the percentage of CD14^+^HLA-DR^−/low^ MDSCs in the patients correlate with CRLM

**Fig. 7:**
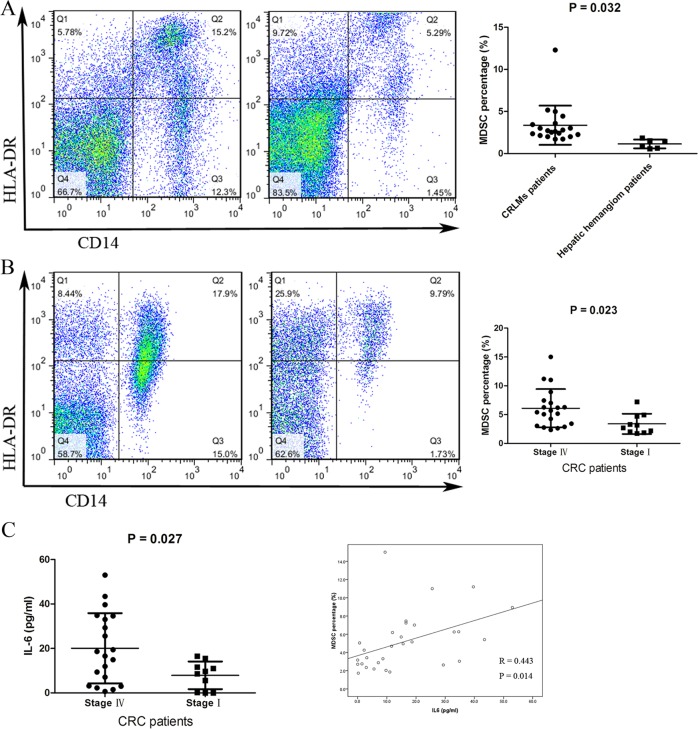
High level of IL-6 associated with MDSCs in the patients correlate with CRLM. **a** The levels of MDSCs in paracancer liver tissue obtained from CRLM patients (*n* = 20) and hepatic hemangioma patients (*n* = 6) were measured using FACS. **b** The levels of MDSCs in plasma samples obtained from CRLM patients (*n* = 20) and stage I CRC patients (*n* = 10) were measured using FACS. **c** IL-6 l evels in plasma samples obtained from CRLM patients (*n* = 20) and stage I CRC patients (*n* = 10) were measured using ELISA. The correlation analysis was performed between IL-6 levels and MDSCs percentages from the pooled CRC patients (*n* = 30) (^*^P < 0.05, ^**^P < 0.01)

In the CRLM mouse model, high levels of IL-6 and associated MDSCs stimulated by the S1PR1–STAT3 signaling pathway correlated with the number of liver metastatic nodes (Fig. 5). We then reevaluated the results in CRLM patients. We found that the percentage of CD14^+^HLA-DR^−/low^ MDSCs in the paracancer liver tissue of CRLM patients (*n* = 20) was higher than the hepatic hemangioma patients (*n* = 6) (3.4% ± 2.3% vs. 1.1% ± 0.5%, *P* = 0.032) (Fig. 7a). The percentage of MDSCs (6.1% ± 3.3% vs. 3.4% ± 1.8%, *P* = 0.023) (Fig. 7b) and the level of circulating IL-6 (20.1 ± 15.8 pg/ml vs. 7.9 ± 6.2 pg/ml, *P* = 0.027) (Fig. 7c) in the peripheral blood of CRLM patients (*n* = 20) are higher than the stage I CRC patients (*n* = 10). Moreover, there was a strong correlation between the IL-6 level and the percentage of MDSCs in the pooled CRC patients (*R* = 0.443, *P* = 0.014) (Fig. 7c).

### Increased CD14^+^HLA-DR^−/low^ MDSCs from paracancer liver tissue of CRLM patients inhibited autologous T-cell proliferation and predict poor prognosis

**Fig. 8:**
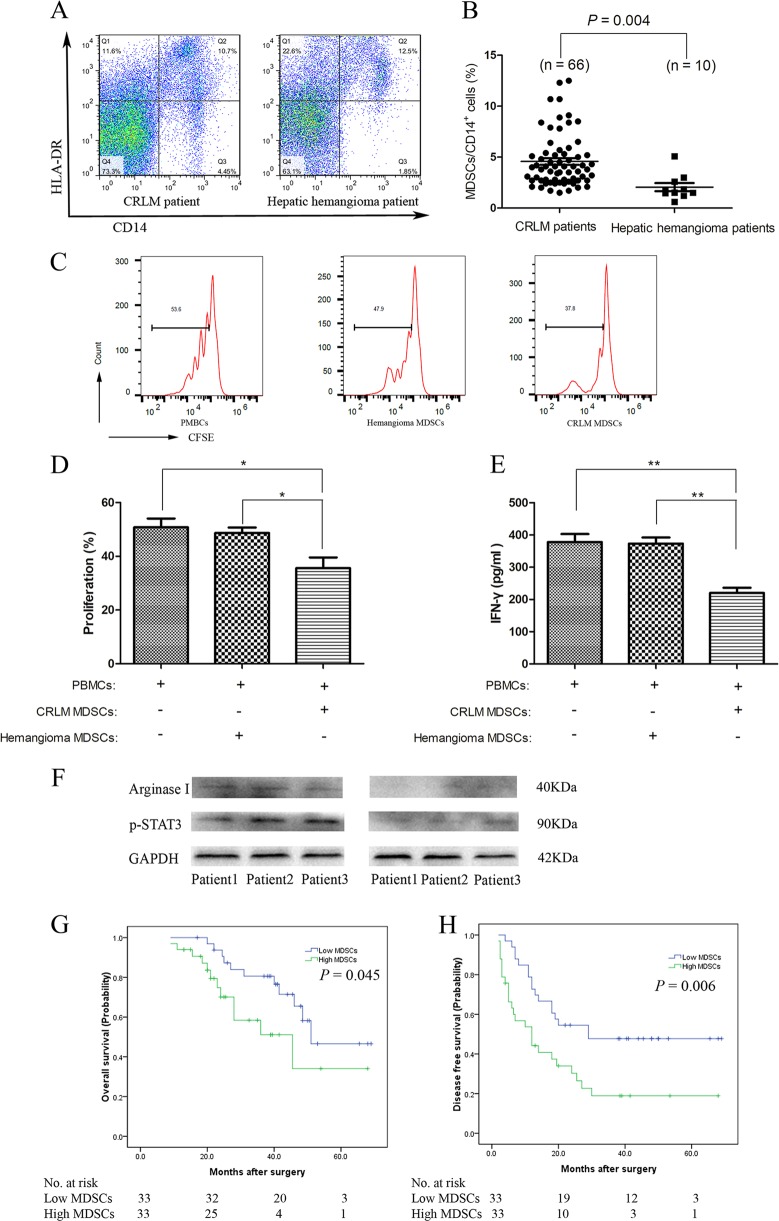
Increased CD14^+^HLA-DR^−/low^ MDSCs from CRLM patients inhibited autologous T-cell proliferation and predict poor prognosis in patients with synchronous CRLM **a** The measurements of CD14^+^HLA-DR^−/low^ MDSCs with Flow cytometry in paracancer liver tissues of CRLM patients compared to those in parahemangioma liver tissues of hepatic hemangioma patients using anti-CD14 and anti-HLA-DR antibodies. **b** The frequency of MDSCs was significantly higher in CRLM patients than in hemangioma patients (*P* = 0.004). **c**, **d** Proliferation of PBMCs after a T-cell activation/expansion kit stimulation for 96 h in MDSCs presence isolated from CRLM and hepatic hemangioma patients was evaluated by the CFSE. CD14^+^HLA-DR^−/low^ MDSCs from CRLM patients significantly decreased the proliferation of PBMC compared to those from hepatic hemangioma patients. **e** CD14^+^HLA-DR^−^^/low^ MDSCs from CRLM patients significantly inhibited T-cell secretion of IFN-γ compared to those from hepatic hemangioma patients (*n* = 6). **f** Increased expression of p-STAT3 and arginase I was detected in the CD14^+^HLA-DR^−/low^ MDSCs from CRLM patients than those in hepatic hemangioma patients by Western blotting. **g** Patients with low-MDSC counts had a longer OS than those with high MDSC counts (*P* = 0.045). **h** DFS was determined for the high-MDSC and low-MDSC groups. Patients with low-MDSC counts had a longer DFS than those with high-MDSC counts had shorter DFS based on the median MDSCs (*P* = 0.006) (^*^P < 0.05; ^**^P < 0.01)

In another cohort CRLM patients, we compared the number of CD14^+^HLA-DR^−^^/low^ MDSCs between the paracancer liver tissue of 66 CRLM patients and 10 hepatic hemangioma patients by flow cytometry, we found the number of CD14^+^HLA-DR^−/low^ MDSCs was significantly higher (4.6 ± 2.6%, *n* = 66) in paracancer liver tissue of CRLM patients than that in hepatic hemangioma patients (2.1 ± 1.3%, *n* = 10) (*P* = 0.004) (Fig. 8a, b). The gating strategy for human MDSCs was showed in Supplementary Fig. 4A, B.

The immunosuppressive activity of CD14^+^HLA-DR^−^^/low^ MDSCs from paracancer liver tissue of CRLM patients and hepatic hemangioma patients was evaluated. Sorted MDSCs were added to peripheral blood mononuclear cells (PBMCs) with autologous anti-CD3/CD28-stimulation, the proliferation and the release of Interferon-γ (IFN-γ) were analyzed by carboxyfluorescein succinimidyl ester (CFSE)-based proliferation assay and enzyme-linked immunosorbent assay (ELISA). Only CD14^+^HLA-DR^−/low^ MDSCs from the paracancer liver tissue of CRLM patients significantly suppressed proliferation (35.58 ± 5.73% vs. 48.65 ± 5.03%, *P* < 0.05; Fig. 8c, d) and IFN-γ production (220.17 ± 39.69 pg/ml vs. 373.50 ± 46.53 pg/ml, *P* < 0.01; Fig. 8e) of autologous PBMCs compared to those of the hemangioma patients. Given that p-STAT3 and arginase I have been reported to be participated in the CD14^+^HLA-DR^−/low^MDSC-mediated T-cell suppression function in several human cancers^16,17^, we compared the p-STAT3 and arginase I expression between these two groups. We found more expression of these two markers in the CD14^+^HLA-DR^−/low^ MDSCs separated from the paracancer liver tissue of CRLM patients compared to those from hemangioma patients (Fig. 8f).

To investigate the prognostic value of high MDSCs in the paracancer liver of CRLM patients, the 66 CRLM patients was stratified into two groups: the low-MDSC (≤3.9%) and the high-MDSC (>3.9%) group, based on the median of the CD14^+^HLA-DR^−/low^ MDSCs in CRLM patients (3.9%). We compared OS between the two groups by a Kaplan–Meier survival analysis. The OS of CRLM patients in the high-MDSC group was significantly worse than that of those patients in the low-MDSC group (*P* = 0.045; Fig. 8g), demonstrating the key impact of MDSCs on the clinical outcome of CRLM patients. Furthermore, we also certified that the DFS for patients in the high-MDSC group was significantly shorter than for patients in the low-MDSC group among the 66 CRLM patients (*P* = 0.006; Fig. 8h). To determine the clinical effect of different prognostic factors that might impair the survival of the study objectives, univariate analyses were used for OS in 66 patients with CRLM. Showed in Table 4, the primary nodal (N) stage (*P* = 0.020), tumor deposits (*P* = 0.048), vascular invasion (*P* = 0.005), nerve invasion (*P* = 0.012), and being in the high-MDSC group (*P* = 0.045) were risk factors influencing the OS of patients with CRLM with significant statistical differences. To investigate the robustness of the prognostic effect of having a high frequency of MDSCs, Cox multivariate regression analyses were used to derive the independent risk estimates about OS with the covariates that showed significance in the univariate analyses. The primary nodal (N) stage (*P* = 0.047), vascular invasion (*P* = 0.010), and having a high frequency of MDSCs (*P* = 0.022) were identified as independent prognostic factors for OS in the 66 patients with CRLM (Table 4). With the same statistical methods, tumor deposits (*P* = 0.018), vascular invasion (*P* = 0.030), and having a high frequency of MDSCs (*P* = 0.004) were identified as independent prognostic predictor for DFS (Table 5). To summary, our results demonstrated that high MDSCs numbers could be a useful predictor for the survival of CRLM patients.

**Table 4:** Univariate and multivariate analyses of the associations between OS and the clinicopathological characteristics of the 66 CRLM patients who underwent simultaneous R0 resection

Prognostic factor	Univariate analysis			Multivariate analysis		
	HR	95% CI	*P*	HR	95% CI	*P*
Age (>60:≤60)	1.434	0.610–3.370	0.409			
Sex (female:male)	1.847	0.809–4.216	0.145			
Primary tumor site (left:right)	0.762	0.299–1.937	0.568			
Histological type (mucinous adenocarcinoma: adenocarcinoma)	1.442	0.530–3.921	0.473			
Tumor differentiation (well, moderate: poor and other)	2.494	0.738–8.423	0.141			
Primary tumor (T) stage (T3,T4:T1,T2)	0.459	0.134–1.578	0.216			
Primary nodal (N) stage (N1,N2:N0)	3.686	1.231–11.044	0.020	3.130	1.014–9.662	0.047
Tumor deposits (positive: negative)	2.411	1.009–5.764	0.048	1.661	0.592–4.661	0.336
Vascular invasion (positive:negative)	3.303	1.432–7.621	0.005	3.150	1.311–7.569	0.010
Nerve invasion (positive:negative)	2.933	1.273–6.762	0.012	2.070	0.860–4.981	0.104
No. of LMs (≥4:≤3)	1.391	0.470–4.120	0.551			
Size of LM (≥5 cm:<5 cm)	0.550	0.186–1.626	0.280			
CEA (≥5 ng/ml:<5 ng/ml)	1.828	0.675–4.949	0.235			
Tumor distribution (bilobar: unilobar)	1.647	0.664–4.083	0.282			
*KRAS* status (mutated: wild type)	1.816	0.782–4.221	0.165			
MDSCs (high: low)	2.316	0.997–5.382	0.045	2.732	1.153–6.476	0.022

**Table 5:** Univariate and multivariate analyses of the associations between the DFS and the clinicopathological characteristics of the 66 CRLM patients who underwent simultaneous R0 resection

Prognostic factor	Univariate analysis			Multivariate analysis		
	HR	95% CI	*P*	HR	95% CI	*P*
Age (>60:≤60)	0.683	0.369–1.266	0.266			
Sex (female:male)	1.342	0.705–2.554	0.370			
Primary tumor site (left:right)	0.994	0.475–2.077	0.986			
Histological type (mucinous adenocarcinoma: adenocarcinoma)	1.116	0.495–2.520	0.791			
Tumor differentiation (well, moderate: poor and other)	1.842	0.852–3.985	0.121			
Primary tumor (T) stage (T3,T4:T1,T2)	0.971	0.299–3.148	0.960			
Primary nodal (N) stage (N1,N2:N0)	2.214	1.056–4.644	0.035	1.640	0.742–3.623	0.221
Tumor deposits (positive: negative)	2.404	1.280–4.518	0.006	2.200	1.143–4.236	0.018
Vascular invasion (positive:negative)	2.272	1.202–4.293	0.012	2.084	1.073–4.046	0.030
Nerve invasion (positive:negative)	2.058	1.108–3.823	0.022	1.412	0.717–2.782	0.318
No. of LMs (≥4:≤3)	1.853	0.851–4.035	0.120			
Size of LM (≥5 cm:<5 cm)	0.930	0.457–1.892	0.841			
CEA (≥5 ng/ml:<5 ng/ml)	1.289	0.656–2.534	0.462			
Tumor distribution (bilobar: unilobar)	1.358	0.681–2.709	0.385			
*KRAS* status (mutated: wild type)	1.674	0.878–3.192	0.118			
MDSCs (high: low)	2.313	1.244–4.300	0.008	2.513	1.346–4.693	0.004

## Discussion

In this study, we showed that a mutual activation loop between S1PR1 and STAT3 can enhance CRC cell proliferation, migration and invasion in vitro and in vivo. p-STAT3 was the dependent signaling pathway of S1PR1 in the promotion of cell growth and liver metastasis in CRC. The level of IL-6 and the associated MDSCs stimulated by the S1PR1–STAT3 signaling pathway correlate with the number of liver metastatic nodes in the CRLM mouse model and CRLM patients. Furthermore, we found that MDSCs formed a premetastatic niche in the liver that can prime the distant organ microenvironment. Increased CD14^+^HLA-DR^−/low^ MDSCs in the paracancer liver tissue of CRLM patients inhibited autologous T-cell proliferation and predict poor prognosis.

S1PR1 is one of the five G protein-coupled receptors for sphingosine-1-phosphate (S1P), and it is crucial for the retention of lymphocytes in secondary lymphoid organs^18,19^. S1PR1 is also highly expressed in endothelial cells and pericytes, and the expression of S1PR1 within these cells is important for tumor angiogenesis and metastasis^20,21^. Blocking S1PR1 can retain lymphoma cells in lymphoid organs because of an egress function defect^22,23^. STAT3 is a transcription factor for S1PR1 and, simultaneously, enhanced S1PR1 expression activates STAT3 and upregulates IL-6 expression, a proinflammatory cytokine crucial for STAT3 activation and inflammatory cell-mediated transformation and tumor progression. Thus, this is a new feed forward mechanism that explains the persistent STAT3 activation in cancer cells and the tumor microenvironment, which is important for malignant tumor progression and metastasis^3^. Recently, very few studies found over co-expression of S1PR1 and p-STAT3 in activated B cell-like diffuse large B-cell lymphoma^13,24^ and a correlation with poor prognosis^24^. Our study was the first designed to investigate the expression and prognostic significance of S1PR1 and p-STAT3 expression in human CRC.

Another discovery in our study was that IL-6 changed most significantly among p-STAT3 downstream proteins when S1PR1 was over or lowly expressed in CRC cell lines. Recently, tumor-secreted IL-6 has been reported to stimulate MDSCs generation and accumulation^5,6^. Therefore, we hypothesized that IL-6 may play a critical role in the induction of MDSCs in CRLM models and patients. The results confirmed our hypothesis. We found that more IL-6 with more associated MDSCs correlated with the number of liver metastases, not only in mouse models, but also in the CRC patients. This phenomenon was previously shown by other studies in squamous cell cancer of the esophagus^25^ and breast cancer^15^.

In recent years, there has been heightened interest in MDSCs and their biological function in tumor pathobiology. MDSCs can potently suppress T-cell function through a number of mechanisms, modulate the activity of NK and myeloid cells, induce regulatory T cells, etc.^5–7,26,27^. A significant correlation was reported among circulating MDSCs, metastatic burden, clinical stage, chemotherapy effect, and tumor refractoriness to anti-VEGF treatment in some human cancers, including CRC^5–7,26,27^.

MDSCs were originally identified in tumor-bearing mice as cells that co-express CD11b and GR1, subpopulations have been shown to exist: polymorphonuclear (PMN)-MDSC (CD11b^+^Ly6G^+^Ly6C^lo^) and monocytic (M)-MDSC (CD11b^+^Ly6G^-^Ly6C^hi^); however, their phenotype in human cancer is rather diverse, MDSCs with the LIN^−^HLA^−^DR^−^CD33^+^CD11b^+^^5,26^, CD33^+^HLA-DR^−^^28^ and CD14^+^HLA-DR^−^^/low^ phenotypes^29^ have been isolated from the blood of CRC patients. The CD14^+^HLA-DR^−^^/low^ MDSCs that were detected in our study were also found to be increased in several other human cancers, including clear cell renal cell carcinoma^30^, hepatocellular carcinoma^31–33^, melanoma^34^, head and neck squamous cell carcinoma^16^, nonsmall cell lung cancer;^35^ additionally, an increase in the frequency of CD14^+^HLA-DR^−/low^ MDSCs with suppressor functions in the peripheral blood from patients with inflammatory bowel disease was observed^17^. A report in 2019 demonstrated that there was significant expansion of CD14^+^HLA-DR^−^^/low^ MDSCs in all of included 198 metastatic solid cancers (colorectal, *n* = 64; lung, *n* = 20; bladder, *n* = 4; breast, *n* = 11; kidney, *n* = 12; melanoma, *n* = 25; pancreatic, *n* = 37; prostate cancer, *n* = 25) tested compared with healthy donors (*P* < 0.03)^36^.

We initially used the marker LIN^−^HLA^−^DR^−^CD33^+^CD11b^+^ for the CRC patients defined in the above studies^5,26^, but the percentage was too small to obtain reasonable and statistical results. Therefore, the controversy regarding the phenotype in human cancers has not been resolved until the publication of the recommendations for MDSCs nomenclature and characterization standards in 2016^37^. It proposes an algorithmic approach that first focuses on the phenotypic characterization of the cells, and presence of suppressive activity would define these cells as MDSCs in mice and humans. In human PBMC, the equivalent to PMN-MDSC are defined as CD11b^+^CD14^−^CD15^+^ or CD11b^+^CD14^−^CD66b^+^ and M-MDSC as CD11b^+^CD14^+^HLA-DR^−/lo^CD15^–37^. The limitation of our study is that we accomplished MDSCs detection from January 2013 to December 2015 before the publication of the recommendations in 2016 by Bronte et al.^37^, we will follow the recommendations in subsequent experiment.

Interestingly, many tumors show a metastatic predisposition to selected organs, such as liver cancer to lung, breast cancer to liver and bone, and CRC to the liver. Organ tropism, more classically known as the seed-and-soil hypothesis, was first proposed by Stephen Paget in 1889, when he concluded that the distribution of metastases was not random but instead displayed clear organ preference. Genes that are responsible for the acquisition of metastatic abilities, metastatic tissue tropism, and the nature of metastasis predisposition factors have been partly identified^38^. At the same time, cancer-associated host immune reaction and inflammation are indispensable factors in tumor progression and metastasis^39^. Paget’s hypothesis later led to the idea that before metastatic dissemination, primary tumors secrete factors that contribute to the development of a premetastatic niche, defined by the development of an environment distant from the primary tumor that is suitable for the survival and outgrowth of incoming circulating tumor cells^40^, and can be primed and established through a complex interplay among primary tumor-derived factors, tumor-mobilized bone marrow-derived cells, and local stromal componen^41^. Recent studies underscore the importance of bone marrow-derived hematopoietic progenitor cells and that myeloid cells form a “premetastatic niche” regulating organ-specific tumor spread in the lung^8,9,41–44^ and in the liver^45,46^. However, no study has focused on CRLM, and the mechanisms by which these cells mediate outgrowth of metastatic tumor cells are not completely known. Our study bridged the gap. We found that MDSCs formed the premetastatic niche in the liver that can prime the distant organ microenvironment. Furthermore, we found that S1PR1–STAT3 upregulation in tumor cells induces IL-6, which activated S1PR1–STAT3 in MDSCs in the liver, leading to premetastatic niche formation prior to CRC cell arrival. Our results indicate that tumor-derived factors are initiating effectors of niche formation. If the tumors are removed quickly and completely, there would not be premetastatic niches for therapeutic intervention. However, many CRC patients cannot have their tumors removed quickly and/or completely, causing relapses. Especially with CRLM patients, even after the primary and liver metastatic tumors are radically resected, the niche is still there, and recurrence in the liver is inevitable. Perhaps the most significant aspect of our study is the therapeutic potential to target the S1PR1–STAT3 signaling axis to eliminate and/or reduce preformed premetastatic niches, thereby preventing metastasis. The results from treatment of STAT3 or S1PR1 in the myeloid compartment by CpG-Stat3 siRNA and CpG-S1pr1 siRNA would effectively reduce preformed metastatic niches at distant organs, supporting the notion that targeting STAT3/S1PR1 signaling in immune cells can reduce STAT3 activity and myeloid cell infiltrate in future metastatic sites and thereby prevent metastasis^42^.

## Conclusions

To prevent metastasis by eliminating premetastatic niches is an attractive approach for effective cancer treatment. Our study suggests that the S1PR1–STAT3 axis operates not only in tumor cells but also in MDSCs involved in the promotion of growth and liver metastasis in CRC. We demonstrated that S1PR1–STAT3 is an effective target to disable both tumor cells and “non-neoplastic” cells from creating an environment that is crucial for malignant distant outgrowth.

## Materials and methods

### Selection of patient material

From October 2006 to October 2011, 153 human CRC and paired nontumor tissue samples, which had been formalin-fixed and paraffin-embedded, and clinicopathologic data were retrieved from our prospectively constructed CRC database. Prospective data collection and data quality management were performed by an independent full-time research assistant. None of the patients received any preoperative chemotherapy or radiation. The tumor stage was determined according to the seventh edition of the International Union Against Cancer (UICC)/American Joint Committee on Cancer (AJCC) TNM classification^47^. Routine chemoradiotherapy had been given postoperatively to the patients with advanced-stage disease. The OS was calculated from the day of surgery to the date of death due to CRC or last follow-up. DFS was measured from the date of surgery to the date of documented disease recurrence or metastasis. The clinical information for the CRC patients is presented in Table 1.

We prospectively identified 66 synchronous CRLM patients who were treated with simultaneous curative surgery for initially resectable disease between January 2013 and December 2015. The criteria of selection of simultaneous surgery have been presently published^48^, including the following: the primary disease are to be resected radically and liver metastases are to be R0 (negative margin) resected; extrahepatic metastases are resectable; an adequate volume of future liver remnant postresection; histologically proven CRLM; no other systemic diseases; and not being treated with chemotherapy, targeted therapy or immunotherapy before resection. MDSCs were isolated from the resected livers within 2 h. Routine chemotherapy was given to these patients after the operation.

### Immunohistochemistry and staining evaluation

Immunohistochemistry (IHC) and staining evaluation studies of S1PR1 and p-STAT3 expression were according to a protocol described^13,49^. All of the specimens were pathologically reassessed independently by two gastroenterology pathologists blinded to the clinical data.

### Cell lines, plasmid construction, and transfection

All of the cell lines (Caco-2, DLD-1, HT-29, SW480, SW620, HCT116, LOVO, MC38, and CT26) were obtained from the Chinese Academy of Sciences and cultured in 1640 or DMEM medium with 10% fetal bovine serum (Logan Utah, HyClone, USA). The plasmid expressing mouse S1PR1 was a generous gift from Prof. Hua Yu^3^. The murine pMXs-STAT3C plasmid was acquired from Addgene^50^. Other lentivirus vectors (purchased from Shanghai Genechem Ltd.) for overexpression and shRNA of S1PR1 and STAT3 were successfully constructed; transfection of the vectors was performed according to the manufacturer’s protocol. The protein expression in these cells was examined using Western blotting.

### Assays for tumor growth, invasion, and migration in vitro

The infected cells were used in MTT, transwell and wound healing assays, as described previously^51^.

### Assays for tumor growth and liver metastasis in vivo

The infected cells were used to construct a subcutaneous tumor-bearing and CRLM nude mouse model in vivo^52,53^.

### Measurement of IL-6 in the serum and culture media by ELISA

IL-6 levels in cell culture supernatant and serum of human and murine samples were analyzed using IL-6 human and mouse ELISA kits according to the manufacturer’s protocol.

### Tumor conditioned medium treatment

TCM was digested with trypsin (200 μg/ml) for 2 h, followed by inactivation with trypsin inhibitor (1:1 ratio). The TCM was stored at −80 °C for further experiments^42^.

### MDSCs isolation and flow cytometry analysis

The resected livers were cut into pieces and resuspended with a digestion enzyme mixture (2 mg/ml collagenase I, Invitrogen; 1 mg/mldispaseII, Sigma). The mixture of tissue/enzyme was put in a 37 °C shaker for 30 min and then poured through 70 μm cell strainers (BD Biosciences). After removing the hepatocytes using a Ficoll gradient (Percoll^™^ PLUS/Percoll, GE) to obtain single-cell suspensions, flow cytometry analysis and fluorescence-activated cell sorting was fulfilled using fluorescently labeled antibodies (PE mouse anti-human CD14, PerCP-Cy^™^5.5 mouse anti-human HLA-DR; PerCP-CyTM5.5 rabbit anti-mouse CD11b and APC rabbit anti-mouse Ly-6G and Ly-6C, BD). The PBMCs were separated from the peripheral blood of the patients by density gradient centrifugation (Percoll^™^ PLUS/Percoll, GE)^25,29,54^.

### T-cell suppression assay and cytokine secretion

The T-cell activation/expansion kit (Miltenyi Biotech) was used to stimulate the 10^4^ PBMCs and cultured together with MDSCs at a 1:2 ratio. Proliferation was measured after 96 h by proliferation assays with CFSE following the manufacturer’s instructions (Invitrogen). For the IFN-γ responses evaluation, the cell culture supernatants acquired from the suppression assay were collected after 96 h and evaluated using ELISA (eBioscience) following the instructions of the manufacturer.

### Statistical analyses

Continuous data were measured using a t-test. For categorical data, chi-squared analysis or Fisher’s exact test was used. The survival rate was analyzed with the Kaplan–Meier method, and differences in survival rates were assessed with the log-rank test. A Cox proportional hazards model was used for multivariate analysis. All statistical analyses were performed using SPSS 16.0 software (SPSS, Chicago, IL, USA). Two-sided *P* values were calculated, and *P* < 0.05 was considered significant.

## Supplementary information

Supplementary Materials and Methods

Supplementary Figure 1

Supplementary Figure 2

Supplementary Figure 3

Supplementary Figure 4

## References

[1] Petrelli NJ (2008). Perioperative or adjuvant therapy for resectable colorectal hepatic metastases. J. Clin. Oncol..

[2] Hanahan D, Coussens LM (2012). Accessories to the crime: functions of cells recruited to the tumor microenvironment. Cancer Cell.

[3] Lee H (2010). STAT3-induced S1PR1 expression is crucial for persistent STAT3 activation in tumors. Nat. Med..

[4] Kishimoto T (2005). Interleukin-6: from basic science to medicine-40 years in immunology. Annu. Rev. Immunol..

[5] Gabrilovich DI, Ostrand-Rosenberg S, Bronte V (2012). Coordinated regulation of myeloid cells by tumours. Nat. Rev. Immunol..

[6] Condamine T, Gabrilovich DI (2011). Molecular mechanisms regulating myeloid-derived suppressor cell differentiation and function. Trends Immunol..

[7] Talmadge JE, Gabrilovich DI (2013). History of myeloid-derived suppressor cells. Nat. Rev. Cancer.

[8] Hiratsuka S, Watanabe A, Aburatani H, Maru Y (2006). Tumour-mediated upregulation of chemoattractants and recruitment of myeloid cells predetermines lung metastasis. Nat. Cell Biol..

[9] Yan HH (2010). Gr-1+CD11b+ myeloid cells tip the balance of immune protection to tumor promotion in the premetastatic lung. Cancer Res..

[10] Lin Q (2014). Aberrant expression of sphingosine-1-phosphate receptor 1 correlates with metachronous liver metastasis and poor prognosis in colorectal cancer. Tumour Biol..

[11] Tye H (2012). STAT3-driven upregulation of TLR2 promotes gastric tumorigenesis independent of tumor inflammation. Cancer Cell.

[12] Zhang L (2014). Growth arrest and DNA damage 45G down-regulation contributes to Janus kinase/signal transducer and activator of transcription 3 activation and cellular senescence evasion in hepatocellular carcinoma. Hepatology.

[13] Liu Y (2012). S1PR1 is an effective target to block STAT3 signaling in activated B cell-like diffuse large B-cell lymphoma. Blood.

[14] Lesina M (2011). Stat3/Socs3 activation by IL-6 transsignaling promotes progression of pancreatic intraepithelial neoplasia and development of pancreatic cancer. Cancer Cell.

[15] Oh K (2013). A mutual activation loop between breast cancer cells and myeloid-derived suppressor cells facilitates spontaneous metastasis through IL-6 trans-signaling in a murine model. Breast Cancer Res..

[16] Vasquez-Dunddel D (2013). STAT3 regulates arginase-I in myeloid-derived suppressor cells from cancer patients. J. Clin. Investig..

[17] Haile LA (2008). Myeloid-derived suppressor cells in inflammatory bowel disease: a new immunoregulatory pathway. Gastroenterology.

[18] Arnon TI, Horton RM, Grigorova IL, Cyster JG (2013). Visualization of splenic marginal zone B-cell shuttling and follicular B-cell egress. Nature.

[19] Proia RL, Hla T (2015). Emerging biology of sphingosine-1-phosphate: its role in pathogenesis and therapy. J. Clin. Investig..

[20] Chae SS, Paik JH, Furneaux H, Hla T (2004). Requirement for sphingosine 1-phosphate receptor-1 in tumor angiogenesis demonstrated by in vivo RNA interference. J. Clin. Investig..

[21] Visentin B (2006). Validation of an anti-sphingosine-1-phosphate antibody as a potential therapeutic in reducing growth, invasion, and angiogenesis in multiple tumor lineages. Cancer Cell.

[22] Mandala S (2002). Alteration of lymphocyte trafficking by sphingosine-1-phosphate receptor agonists. Science.

[23] Matloubian M (2004). Lymphocyte egress from thymus and peripheral lymphoid organs is dependent on S1P receptor 1. Nature.

[24] Paik JH (2014). Overexpression of sphingosine-1-phosphate receptor 1 and phospho-signal transducer and activator of transcription 3 is associated with poor prognosis in rituximab-treated diffuse large B-cell lymphomas. BMC Cancer.

[25] Chen MF (2014). IL-6-stimulated CD11b+ CD14+ HLA-DR- myeloid-derived suppressor cells, are associated with progression and poor prognosis in squamous cell carcinoma of the esophagus. Oncotarget.

[26] Solito S (2011). A human promyelocytic-like population is responsible for the immune suppression mediated by myeloid-derived suppressor cells. Blood.

[27] Kanterman J (2014). Adverse immunoregulatory effects of 5FU and CPT11 chemotherapy on myeloid-derived suppressor cells and colorectal cancer outcomes. Cancer Res..

[28] Sun HL (2012). Increased frequency and clinical significance of myeloid-derived suppressor cells in human colorectal carcinoma. World J. Gastroenterol..

[29] Duffy A (2013). Comparative analysis of monocytic and granulocytic myeloid-derived suppressor cell subsets in patients with gastrointestinal malignancies. Cancer Immunol. Immunother..

[30] Gustafson MP (2015). Intratumoral CD14+ Cells and Circulating CD14+HLA-DRlo/neg Monocytes Correlate with Decreased Survival in Patients with Clear Cell Renal Cell Carcinoma. Clin. Cancer Res..

[31] Hoechst B (2008). A new population of myeloid-derived suppressor cells in hepatocellular carcinoma patients induces CD4(+)CD25(+)Foxp3(+) T cells. Gastroenterology.

[32] Gao XH (2017). Circulating CD14(+) HLA-DR(-/low) myeloid-derived suppressor cells predicted early recurrence of hepatocellular carcinoma after surgery. Hepatol. Res..

[33] Arihara F (2013). Increase in CD14+HLA-DR-/low myeloid-derived suppressor cells in hepatocellular carcinoma patients and its impact on prognosis. Cancer Immunol. Immunother..

[34] Poschke I, Mougiakakos D, Hansson J, Masucci GV, Kiessling R (2010). Immature immunosuppressive CD14+HLA-DR-/low cells in melanoma patients are Stat3hi and overexpress CD80, CD83, and DC-sign. Cancer Res..

[35] Huang A (2013). Increased CD14(+)HLA-DR (-/low) myeloid-derived suppressor cells correlate with extrathoracic metastasis and poor response to chemotherapy in non-small cell lung cancer patients. Cancer Immunol. Immunother..

[36] Kobayashi M (2019). Blocking monocytic myeloid-derived suppressor cell function via anti-DC-HIL/GPNMB antibody restores the in vitro integrity of T cells from cancer patients. Clin. Cancer Res..

[37] Bronte V (2016). Recommendations for myeloid-derived suppressor cell nomenclature and characterization standards. Nat. Commun..

[38] Nguyen DX, Massague J (2007). Genetic determinants of cancer metastasis. Nat. Rev. Genet..

[39] Quail DF, Joyce JA (2013). Microenvironmental regulation of tumor progression and metastasis. Nat. Med..

[40] Becker A (2016). Extracellular vesicles in cancer: cell-to-cell mediators of metastasis. Cancer Cell.

[41] Liu Y (2016). Tumor exosomal RNAs promote lung pre-metastatic niche formation by activating alveolar epithelial TLR3 to recruit neutrophils. Cancer Cell.

[42] Deng J (2012). S1PR1-STAT3 signaling is crucial for myeloid cell colonization at future metastatic sites. Cancer Cell.

[43] Kaplan RN (2005). VEGFR1-positive haematopoietic bone marrow progenitors initiate the pre-metastatic niche. Nature.

[44] Clever D (2016). Oxygen sensing by T cells establishes an immunologically tolerant metastatic niche. Cell.

[45] Steele CW (2016). CXCR2 inhibition profoundly suppresses metastases and augments immunotherapy in pancreatic ductal adenocarcinoma. Cancer Cell.

[46] Costa-Silva B (2015). Pancreatic cancer exosomes initiate pre-metastatic niche formation in the liver. Nat. Cell Biol..

[47] Sobin, L. H., Gospodarowicz, M. K., Wittekind, C. & International Union against Cancer. *TNM Classification of Malignant Tumours* 7th edn (Wiley-Blackwell, 2009).

[48] Xu Jianmin, Fan Jia, Qin Xinyu, Cai Jianqiang, Gu Jin, Wang Shan, Wang Xishan, Zhang Suzhan, Zhang Zhongtao (2018). Chinese guidelines for the diagnosis and comprehensive treatment of colorectal liver metastases (version 2018). Journal of Cancer Research and Clinical Oncology.

[49] Kusaba T (2005). Expression of p-STAT3 in human colorectal adenocarcinoma and adenoma; correlation with clinicopathological factors. J. Clin. Pathol..

[50] Takahashi K, Yamanaka S (2006). Induction of pluripotent stem cells from mouse embryonic and adult fibroblast cultures by defined factors. Cell.

[51] Zhang B (2010). Antimetastatic role of Smad4 signaling in colorectal cancer. Gastroenterology.

[52] Zhao L (2013). Recruitment of a myeloid cell subset (CD11b/Gr1 mid) via CCL2/CCR2 promotes the development of colorectal cancer liver metastasis. Hepatology.

[53] Lim SY (2015). Cd11b(+) myeloid cells support hepatic metastasis through down-regulation of angiopoietin-like 7 in cancer cells. Hepatology.

[54] Ilkovitch D, Lopez DM (2009). The liver is a site for tumor-induced myeloid-derived suppressor cell accumulation and immunosuppression. Cancer Res..

