# Supplementary material for: The mechanism of the premetastatic niche facilitating colorectal cancer liver metastasis generated from myeloid-derived suppressor cells induced by the S1PR1–STAT3 signaling pathway
Source: Cell Death Dis. 2019 Sep 18;10(10):693. doi: 10.1038/s41419-019-1922-5 (PMC6751205; doi:10.1038/s41419-019-1922-5)
Supplement: Supplementary file 1 — Supplementary Materials and Methods [file 41419_2019_1922_MOESM1_ESM.docx]

**Supplementary Materials and Methods**

**Western Blot Analyses** Cells were harvested and scraped in RIPA buffer (20 mM Tris pH 7.5, 150 mMNaCl, 1% Nonidet P-40, 0.5% sodium deoxycholate, 1 mM EDTA and 0.1% SDS) containing 10% protease inhibitor cocktail to obtain the total protein content. The BCA method was used to measure the protein concentration. Each sample was fractionated using 10% SDS-PAGE and blotted onto PVDFmembranes. The membranes were incubated in 5% nonfat dry milk to block nonspecific binding and then blotted with a primary antibody overnight at 4°C. After washes with TBST and incubation with anti-rabbit horseradish peroxidase-conjugated secondary antibody (Biosynthesis Biotechnology, China) for 2 hours at room temperature, the immunocomplexes were visualized using chemiluminescence (GE, USA) according to the manufacturer’s protocol.

**MTT Assay** The proliferation of transfected cells was measured using an MTT assay. A total of 10 μL of MTT (5 mg/mL; Sigma) was added to each well for a final volume of 100 μL of culture medium containing viable cells. After an additional incubation of 4 hours, the resulting formazan was dissolved in 100 μL of isopropanol with 40 mM hydrochloric acid. Spectrophotometric absorbance at 570 nm (for formazan dye) was measured with absorbance at 630 nm as a reference.

**Cell Invasion (transwell) Assay** The invasive ability of CRC cells was determined using Matrigel (BD Pharmingen) -coated 24-well transwell chambers with upper and lower culture compartments separated by polycarbonate membranes with an 8-μm pore (Costar, New York, NY, USA). The bottom chamber was filled with Dulbecco’s modified Eagle’s medium containing 10% fetal bovine serum as a chemoattractant. The transfected cells (5×10^4^) were seeded onto the top chamber and incubated at 37°C in 5% CO2 humidified air for 24 hours. The cells that migrated to the underside of the membrane were stained with Giemsa (Sigma) and counted with a microscope (Olympus, Tokyo, Japan).

**Cell Migration (wound healing) Assay** The cells were seeded onto a 6-well plate, grown to confluence and then starved for 24 hours. Then, a linear wound was formed by scraping a pipette tip across the cell culture. Cell motility in terms of wound closure was measured by photographing 3 random fields 36 hours after wounding.

**Assays for Tumor Growth and Metastasis in Vivo** Six-week-old male athymic nude mice were purchased from the Shanghai Experimental Animal Center. The nude mice were injected subcutaneously in the armpit with 2×10^6^ cells. The tumor volumes were monitored at the indicated times and calculated according to the following formula: 0.5×length×width^2^. The *in vivo* liver metastatic capability of the cells was evaluated in athymic nude mice (n=6 per group) as described previously. Briefly, a small left abdominal incision was made under sterile conditions, and the spleen was exposed. A total of 10^6^ cells in 0.1 mL PBS were injected into the spleens using a sterile syringe with a 27-gauge needle. Ten minutes after the injection, the abdomen was closed. After 6 weeks, the animals were euthanized, and the liver metastases were examined.

**Measurement of IL-6 in the Serum and Culture Media by Enzyme-Linked Immunosorbent Assay (ELISA)** Plasma was collected using EDTA as an anticoagulant and centrifuged at 2000 rpm for 10 minutes to remove cells. One million cells were seeded onto 100-mm dishes and grown for 5 days before the culture media was collected and centrifuged at 2000 rpm for 3 minutes to remove cells. Clear plasma or supernatant was collected to perform ELISA following the manufacturer’s instructions. Briefly, 100μL assay diluent was added into each well, followed by the addition of 100 mL standards and/or samples into each well and incubation at room temperature for 2 hours. After the plate was washed 4 times, 200 mL IL6 conjugates was added into each well and incubated at room temperature for another 2 hours. After 4 washes, 200 mL substrate solution was added into each well and incubated in the dark for 20 minutes before 50 mL of stop solution was added into each well. The plate was read at 450 nm with a wavelength correction at 540 nm.

**Supplementary Figure legends**

**Supplementary Figure 1**

The expression of S1PR1 and p-STAT3 in human CRC cell lines (Caco-2, SW620, LoVo, HCT116, HT-29, SW480 and DLD-1).

**Supplementary Figure 2**

The expression of S1PR1 and p-STAT3 in murine CRC cell line of MC38 and CT26.

**Supplementary Figure3**

The inhibition function of myeloid-derived suppressor cells (MDSCs) from mice models. (A) Proliferation of peripheral blood mononuclear cells (PBMCs) stimulated with a T Cell Activation/Expansion Kit in the presence of MDSCs was measured by the carboxyfluorescein succinimidyl ester assay (CFSE). (B) The MDSCs from paracancer liver tissue of the CRLM mice models (both MC38 and CT26) significantly inhibited the T cell proliferation compared to that from the control liver tissue (**P <* 0.05, ***P <* 0.01).

**Supplementary Figure4**

The gating strategy for MDSCs. (A, B) CD14^+^HLA-DR^-/low^ for human MDSCs and (C) CD11b^+^GR1^+^ for mice MDSCs.
