# Supplementary figures and images for: The mechanism of the premetastatic niche facilitating colorectal cancer liver metastasis generated from myeloid-derived suppressor cells induced by the S1PR1–STAT3 signaling pathway
Source: Cell Death Dis. 2019 Sep 18;10(10):693. doi: 10.1038/s41419-019-1922-5 (PMC6751205; doi:10.1038/s41419-019-1922-5)

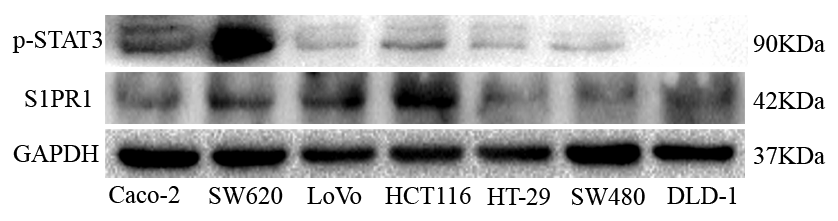

Supplement: Supplementary file 2 — Supplementary Figure 1 [file 41419_2019_1922_MOESM2_ESM.tif]

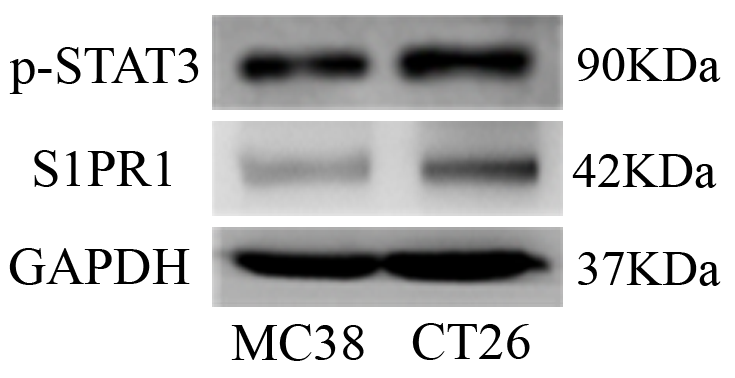

Supplement: Supplementary file 3 — Supplementary Figure 2 [file 41419_2019_1922_MOESM3_ESM.tif]

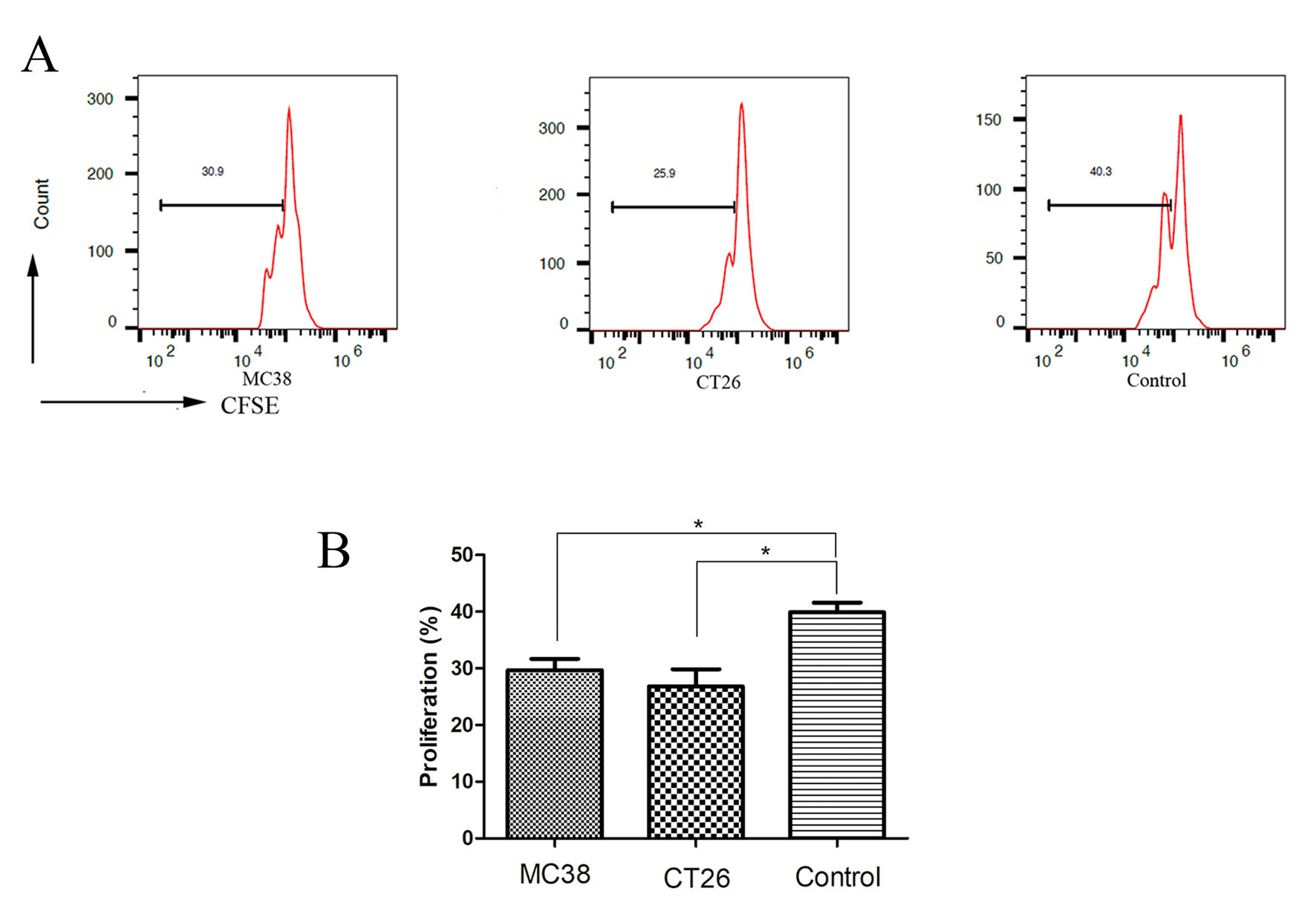

Supplement: Supplementary file 4 — Supplementary Figure 3 [file 41419_2019_1922_MOESM4_ESM.tif]

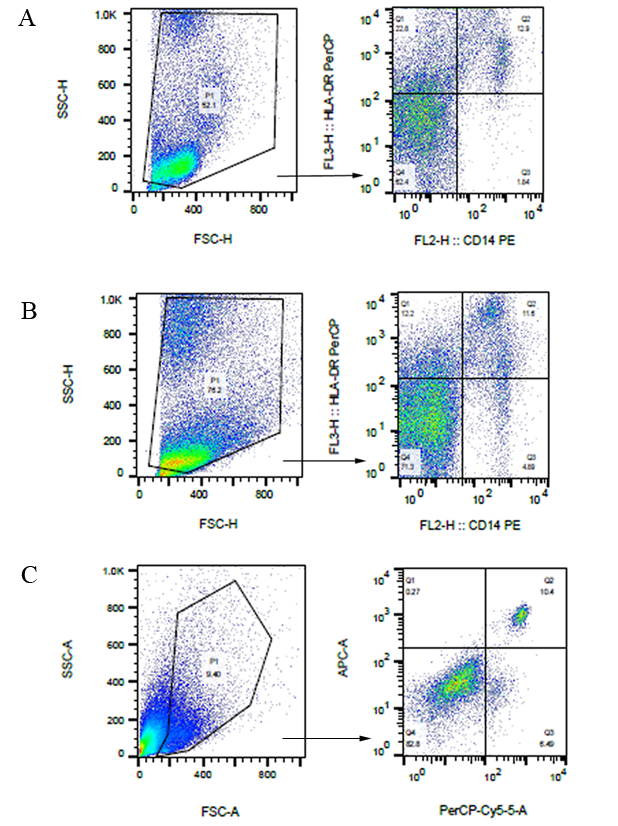

Supplement: Supplementary file 5 — Supplementary Figure 4 [file 41419_2019_1922_MOESM5_ESM.tif]
